# A Qualitative Study of Health Workers' Experiences During Early Surges in the COVID-19 Pandemic in the U.S.: Implications for Ongoing Occupational Health Challenges

**DOI:** 10.3389/fpubh.2022.780711

**Published:** 2022-03-15

**Authors:** Sarah L. Goff, Kate Wallace, Natalia Putnam, Meghan Fernandes, Eva Chow, Marisa DaCosta, Kelsey Clary

**Affiliations:** Department of Health Promotion and Policy, School of Public Health and Health Sciences, University of Massachusetts, Amherst, MA, United States

**Keywords:** health worker, COVID-19, occupational health and safety, qualitative, mitigation

## Abstract

**Background:**

Health workers (HWs) have faced significant threats to physical and psychological health during the COVID-19 pandemic. The recent surges associated with the spread of the delta variant in the U.S., coupled with political resistance to effective public health mitigation strategies, indicate that the risks experienced early in the pandemic are not likely to abate soon. This study sought to better understand the experiences, thoughts, concerns, and recommendations of HWs during one of the first major surges in the U.S. and to explore how these experiences might inform efforts to mitigate potential ongoing COVID-related negative health and psychological impacts on HWs.

**Methods:**

HWs were recruited using a multi-faceted approach tailored to public health mitigation guidelines. Semi-structured interviews were conducted via video conference with front line HWs, support staff, and opioid use disorder service organization providers between April 1 and July 9, 2020 using the Social-Ecological Model as a framework. Interviews were audio-recorded and professionally transcribed; transcripts were analyzed inductively and deductively using thematic analytic methods, generating major themes and subthemes.

**Results:**

A total of 22 HWs participated in the study; 14 were female; 3 identified as a member of a racial or ethnic minority population. Major themes identified included: (1) Institutions, Infrastructure, and the Pandemic; (2) Working Under Fire; (3) The Political Becomes Personal and (4) Hope. Themes and subthemes explicated the ways in which phenomena at personal, interpersonal, community, organizational, and societal levels affected HWs experiences and suggested potential mechanisms through which negative effects on HW mental health and health may be mitigated.

**Conclusions:**

Previous global infectious disease epidemics have had profound negative effects on HWs' health and mental health. This study suggests the potential for similar negative impacts that may be exacerbated by the U.S.'s current sociopolitical milieu. Efforts to systematically describe and quantify these effects and to intervene to mitigate them are warranted.

## Introduction

As of September 9, 2021, COVID-19 had sickened more than 222 million people globally and killed more than 4.6 million (1). The U.S. currently has one of the highest number of cases per 100,000 people in the world and despite having only 4% of the world population, has accounted for an estimated 14% of deaths worldwide (2). New York and Massachusetts experienced major surges in COVID-19 cases in the early days of the U.S. pandemic when little was known about the virus's infectivity, its range of clinical manifestations, or effective treatments. The U.S.'s lack of public health infrastructure and the absence of a coordinated federal government response left state governments and health care systems struggling to procure personal protective equipment (PPE), reliable diagnostic tests, and testing supplies. Health systems and health workers (HWs) had to rapidly develop and test new clinical protocols and learn how to function in the face of great uncertainty. These and other challenges placed a substantial strain on health systems, service organizations, and their HWs. Failure to achieve widespread acceptance of effective mitigation strategies such as masking, social distancing, and vaccination, has led to multiple subsequent surges, extending the strain on health systems, service organizations, and HWs.

Studies of previous global infectious disease epidemics showed significant health and mental health consequences for HWs (3–6). A highly publicized suicide of a New York emergency medicine physician on April 26, 2020 was an early warning sign that the COVID-19 pandemic might also have serious consequences for HWs' mental health (7). Although there is currently no systematic approach to calculating the number of excess deaths among HWs related to COVID-19 in the U.S., a report by the National Nurses Union in September 2020 suggested that the excess HW deaths due to COVID-19 was more than 1,700 (8). The Kaiser Health Network, in partnership with The Guardian, has been tracking the number of COVID-19-related HW deaths, estimating ~3,176 excess deaths by mid-January 2021 (9), a number believed to be a substantial undercount due to the lack of robust tracking systems. Some of the earliest studies of the mental health impacts of the COVID-19 pandemic on HWs took place in countries such as China that experienced high infection rates in January and February, 2021. Relying primarily on survey data, these international studies suggested that HWs were experiencing increased rates of depression, anxiety, post-traumatic stress, and burnout (10–15). U.S.-based studies on the impacts of COVID-19 on HWs have largely consisted of surveys focused on mental health and have also demonstrated increased rates of depression, anxiety, and substance use (16, 17).

Prior studies of HWs during the COVID-19 pandemic have provided important descriptions of the potential negative impacts of the pandemic on HWs' mental health. However, a more nuanced understanding of HWs' experiences during the pandemic, including the potential ways in which institutions and social phenomena may affect their experiences, is needed to be able to decrease the occupational risks experienced by HWs for approaching 2 years. This qualitative study aimed to address this gap in knowledge through interviews with U.S. HWs' across multiple health sectors during the earliest surges in the U.S.

## Methods

### Theoretical Framework

Although there were no empiric data on the effects of the COVID-19 pandemic on HWs when this study was undertaken, the SARS epidemics in China and Hong Kong suggested that HWs could be at risk for pandemic-related mental health sequelae (3–6, 18). The existence of the prior experiences provided a rationale for using both inductive and deductive approaches to studying HWs' COVID-19 experiences. The Social-Ecological Model (SEM) served as the theoretical framework for the study (19) and data collection and analysis were structured to allow for new theory to emerge (20). We selected the SEM because it posits that human development and behavior are influenced by nested individual, interpersonal, community, organizational and broader societal levels of influence. The model has been used extensively in public health and health care as an explanatory model and as an intervention framework. Part of the model's applicability for this study lies in its reflection of the nuances of complex systems of human health. The earliest days of the COVID-19 pandemic suggested that geopolitical, national and state governments, health care systems, community and individual factors would shape and be shaped by the pandemic. The SEM's inclusion of interrelated micro-, meso-, and macro-level social constructions makes it an apt theoretical framework for exploring nuances of HW's experiences with the COVID-19 pandemic.

### Study Population and Recruitment

HWs participated in semi-structured interviews conducted via Zoom between April 1 and July 9, 2020. HWs were defined broadly as nurses, medical assistants, clerical staff, janitorial and food service staff, social workers, physicians, pharmacists and pharmacy technologists, psychologists, and community-based substance use service providers to be able to capture a broad range of experiences. A multimodal approach to recruitment was used in response to restrictions related to COVID-19 and anticipation that health workers might have limited availability. The first wave of recruitment included a convenience sample of HWs who were known professionally or personally by research team members and who worked in states experiencing current or recent surges. In the second wave of recruitment, leaders of a community-based coalition of organizations that provide services for people with opioid use disorder (OUD) in Massachusetts partnered with the research team to send an invitation to members on its list serv. One of the 16 people recruited during the first phase ultimately was unable to participate in an interview because they were too busy. Seven were recruited in the second wave; the total number on the list serv was not known. All interviewees were also asked to suggest additional HWs to interview (snowball sampling) (21) and three of the participants were recruited through this method. A letter of invitation that included the purpose of the study, details of what would be asked of participants, and contact information for the investigative team was sent via email with an attached consent form that was reviewed with participants at the start of interview sessions. Zoom interviews were scheduled at a mutually agreed upon time. The study was approved by the University of Massachusetts's Institutional Review Board.

### Interviews

A semi-structured interview guide was developed and pilot-tested for clarity and completeness. Question development was guided by the five SEM levels (individual, interpersonal, community, organizational, societal). The interview guide consisted of open-ended questions with probes related to key areas of interest, including the personal effects of social distancing/quarantine, family and community effects, organizational factors related to the participants' experiences, trusted sources of information, opinions regarding government responses, and positive impacts of the pandemic. The interview guide was amended in an iterative process to add probes as new concepts emerged during interviews. Interviews were conducted by SG, who has extensive experience with qualitative research methods (22–28) including studies in which she trained and supervised students and research assistants, as she did for the current study (KW, MF, NP, EC, and KC), and KC. Interviews were conducted via Zoom using audio and video in compliance with social distancing and travel restrictions in Massachusetts at the time. Interviews were conducted until data saturation was reached, defined as no new concepts emerging over three consecutive interviews, and achieved after ~16 interviews. Between 20 and 30 interviews were estimated to be needed to achieve data saturation based on the homogeneity of work setting among participants and the narrow focus of the study. This estimate was based on recommendations in methodological texts and papers as well as publications of similar studies.in journals with high impact factors (29–32). Interviews were audio-recorded and professionally transcribed verbatim and field notes were taken during the interviews.

### Analysis

Interview transcripts were analyzed using thematic analysis, applying a validated rapid analytic technique (33). This approach was utilized because it has been identified as an important method for research questions that address rapidly changing health and public health risks, such as those presented by the COVID-19 pandemic. Four members of the investigative team (KC, MF, EC, NP) reviewed a subset of the transcripts to familiarize themselves with the interview content. SG generated a template of broad themes derived from the interview questions for the first phase of the analysis; an open category was included on the template to allow for inclusion of concepts that may have fallen outside of either the structured portion of the template or the theoretical framework. Using a deductive approach, analysts each read a subset of the transcripts and identified key concepts which were entered on a separate form for each transcript. With supporting quotations. SG independently analyzed one of each of the other analysts' transcripts to assess agreement; differences in key concepts were resolved through discussion with the full team. In Phase 2 of the analysis, SG identified unifying themes across the key concepts identified in Phase 1 and applied an inductive approach to theorize connections between SEM levels. These unifying themes and connections were discussed with the full team and revised based on discussion, resulting in a comprehensive set of major themes and subthemes. A summary of themes was sent to participants for review and comment (member checking). During the analysis, reflexivity was considered and discussed. Considerations included that SG is a HW (primary care pediatrician and internist), a parent of school-aged children, and teaches an undergraduate-level elective on the U.S. opioid epidemic. EC, MF, NP, and KW had SG as a professor in an elective course in the spring semester of 2020, KC was a master's student advisee of SG's, and MD is an undergraduate research assistant working with SG.

## Results

Of the 22 HWs interviewed, 14 identified as female; 1 as Black, 2 as South Asian, and 18 as Caucasian/white; 7 were physicians and 6 worked in organizations serving people with opioid use disorder (OUD) (Table 1). Concepts pertinent to the study's aims were identified at all SEM levels. Major themes are organized beginning with the outermost context of the SEM (Societal), such as government and state responses, and moving to the innermost (Interpersonal and Personal), such as impact on families, with a final broad theme. The interconnectedness of the SEM levels means that subthemes for each major theme often touch on multiple levels. Major themes included: (1) Institutions, Infrastructure, and the Pandemic (Societal level); (2) Working Under Fire (Organizational, Community, Interpersonal, and Personal levels); (3) The Political Becomes Personal (Interpersonal and Personal levels); and (4) Hope (Societal, Organizational, and Community levels). Major themes and subthemes are described in detail below with supporting quotations. Additional quotations are located in Table 2. Participants' roles in the health care system and a unique study identifier are included in parentheses after quotations; additional details were not attributed to quotations to protect participant confidentiality.

**Table 1:** Participant demographic characteristics.

**Characteristics (*N* = 22)**	***n* (%)**
Average age (range)	47.6 (21–74)
**Self-identified gender**	
Female	14
Male	8
**Self-identified Race/ethnicity**	
Caucasian/White/Western European	18
South Asian/Asian American	2
Black/African American	1
Not reported	1
**State**	
New England (Massachusetts and Rhode Island)	18
New York	3
Colorado	1

**Table 2:** Selected supplementary illustrative quotations.

**Themes and Subthemes**	**Illustrative Quotations**
**1. Institutions, infrastructure, and the pandemic**	
*Federal and state government responses: coordination confusion*	I think the federal government's response has been very concerning. We started with a government that didn't believe in science and I don't think we should be surprised that this government's biggest challenge is science-based… it's painful to watch the federal government not understand science, not listen to scientists, and then [try] to address what's going on. (ED physician/researcher, SG7) I was absolutely shocked when I found out that the CDC and the WHO were not on the same page with wearing masks. … They were giving basically opposite... recommendations. And... I was shocked. I mean they're both organizations that I have extremely high regard for, and I couldn't believe that they were giving differing viewpoints on mask wearing for COVID. (OUD treatment services manager, KC1)
*The syndemic of misinformation*	I think one of the major missteps is giving medical advice if you're not in an actual position [to]. So, for example the hydroxychloroquine azithromycin, or as Trump called it, H and A on Twitter. Recommending that based off of six patients who got the combination in a non-controlled study… these things come up on a daily basis where… government officials are giving advice that is beyond what they should be giving… (ED physician, SG6)
*Rules of the Game*	The government is not going to make [COVID-19] tests, right?... So whether you use the Defense Production Act or not, whether or not you say the right things and create the incentive structures [matters]…a strategic level will make the private sector do what the private sector does... form follows finance. (ED physician/administrator, SG2)
**2. Working under fire**	
*The myth of the health worker hero*	Some people are saying that none of this is true, they're making it more of a big thing than it really is… but… we do have a very bad virus out there that's very contagious… every day a lot of people are dying. (Physical therapist, MF3) … the people [HWs] are putting their lives on the line. And I think society and our federal government colludes with [belief that HW should be expected to take on all risks]… We [HWs] don't make a decision when we enter medicine to throw our lives at risk. We make a decision to… take care of our patients and do our best. We don't sign an oath to torture ourselves for other people ‘cause it… doesn't work… that is a total delusion and it's a misconception. And I think we're learning that through this. It doesn't work. (Psychiatrist/researcher, SG9)
*Changes in clinical practice*	So it's been really cool seeing how… we can if we need to change by the hour, or minute by minute or day by day…we could do it…I never felt like we were losing control of the ship. (ED physician, SG8)
*Organizational leadership*	… it almost feels like there's more of an expectation [from supervisor] to get all of your work done and more… I'm being asked to do... random projects, collect data for random reasons. (OUD treatment services manager, KC1)
*Work-related worry, fear, and loss*	…at my place [nursing home], they [supervisors] are against testing people [residents in the nursing home] it's like they don't want to be branded as the COVID home that has patients with COVID… [If a nursing home worker tests positive] as long as we are asymptomatic… we can go to work… yup, they won't make us quarantine.” (Physical therapist, MF3)
Vulnerable populations	I think the biggest thing with all of this is not even in my little home here, but it's all these people losing their jobs and not putting food on the table. I mean that's the stuff that really scares me. (Nurse practitioner RJ3) … [COVID-19] exacerbated … homelessness… I think some people who are … unstably housed or … couch-surfing, … people are … [saying to them], “no, … you can't stay in my house anymore because I'm worried about … [getting COVID-19]. (OUD treatment coordinator, KC3) I do a lot of work connecting with the jails and working with people … getting out.... there were more people getting out, and that … made people very vulnerable, … especially if you're having to leave jail, and you're going in the street. I mean, are you better off [out of jail during pandemic]]? I don't know. (OUD treatment program director, KC4)
Foregone care	… [in] EDs all over the country, the volume is way down except for COVID. And there's this sort of constant conversation, that's like, ‘Where are the heart attacks?' Like people are just hanging out at home with their heart attacks… we all have stories of people who are in severe pain and wait days and days and days and then finally come in and they are so far along in their disease process that it makes it much, much harder to treat them or save them… (ED physician/researcher, SG7)
Loss of personal connection to patients	… addiction is a disease of isolation, and people need face-to-face. Zoom is fine, and it serves its purpose, but it's not the same as sitting a few feet away from someone who's listening to you intently and understanding where you're coming from. (OUD treatment coordinator, KC7) I know that being a patient and not being able to see your nurse or doctor's face under all of that equipment is also emotionally scarring. (Pharmacy technician, EC3)
**3. The political becomes personal**	
*Health workers as a threat to the public's health*	… it occurred to me that if people who were socially isolated right now who are not working in health care wanted to all get together, they could really do that… if you've been legit isolated for five weeks, go have a party with people who have been legit isolated… but I can't come to your party. (ED physician/researcher, SG7)
*An emotional toll*	… most of the people that I work with have been having lots of anxiety... at night… we all feel like we are coming down with it… we'll feel like we have to clear our throats… like you're going to cough. You feel the same sense of anxiety like you are coming down with it. (Medical records staff, MF5) …it's like a roller coaster… there will be times when you just feel normal, and then there's other times when you're worried about the end of the world… and then there's other times where you're just despondent because you don't know what to do next. (ED physician/researcher, SG7)
*Family: disruptions and silver linings*	... I think the good thing about it being home with them [children] has been that...I can understand what's happening in their lives at school in a very different way than I ever have before… I knew what was happening but being at home seeing that pattern has been extraordinarily different because… I just get him [son] more. (Psychiatrist/researcher, SG9)

### Theme 1: Institutions, Infrastructure, and the Pandemic

Many HWs commented on the role of institutions and infrastructure in combatting the pandemic. The phenomena described in this theme were located primarily at the SEM Societal level. Subthemes included: (1) Federal, and State Government Responses: Coordination Confusion; (2) The Syndemic of Misinformation; and (3) Rules of the (Capitalist) Game. Included in these subthemes are perspectives on how international and domestic political and public health institutions shaped the early arc of the pandemic, and how institutions' responses shaped HWs' and others' perceptions of the institutions. Some HWs discussed the role of social media and the press in the pandemic response. Many HWs felt the lack of public health infrastructure impacted HWs' experiences early in the course of the pandemic and reflected on institutional failures in response to the pandemic.

#### Federal and State Government Responses: Coordination Confusion

A small number of HWs felt that the problems the U.S. was experiencing in its pandemic response were inevitable:

“*I don't know that this could have been avoided… I don't really have a lot of criticism of the government right now*.” (Nurse practitioner, RJ3)

Most HWs felt that the federal government's response to the pandemic was inadequate and a major contributor to the rapidly worsening state of the pandemic in the U.S.

“*... it seems pretty clear that it's [federal government response] not been coordinated… not been systematic. It's been seemingly random, at times brutal, at times… unjust… lacking compassion…It's frustrating to see… states are having to design their own disaster plans, because the power of the federal government is to align disaster response across states… instead, we've got a hodgepodge.”* (Hospitalist, SG1)

One HW with knowledge of the federal government's disaster preparedness programs was puzzled as to why prior plans for such a pandemic were not being implemented.

“*It has been very… hard for me to understand… what came of all the [preparedness] programs... and whether or not they [agencies responsible for programs] were allowed to [be involved in decision-making], during what most people would call the Superbowl [of pandemic preparedness]. We've been talking about it for decades… We have a disinterested health care system. You have a totally underfunded public health system… the knowledge around the pandemic… exists in the military and the National Security Council. It does not exist in health departments.”* (ED physician/administrator, SG2)

Some HWs noted and worried about the loss of trust in the federal government, including agencies such as the Centers for Disease Control and Prevention (CDC).

“*... the erosion of faith in federal leadership and the ability to believe what the CDC was saying…, to believe that there would be an organized response… it is unconscionable and it's impossible for me to get my head around the damage that it has done forever.”* (ED physician, SG3)

In comparison, although some HWs also felt their state governments could have instituted lockdowns sooner, most felt that their governors and state governments had stepped into the leadership void to provide clear, science-based, public health messaging and leadership.

#### The Syndemic of Misinformation

“*… we've got this, like, false information pandemic.” (*ED physician/researcher, SG6*)*

Some HWs felt that it was difficult to obtain reliable up-to-date information about the pandemic due to the rapidly evolving state of knowledge about COVID-19. Others felt the volume of rapidly disseminated misinformation was problematic.

“*We sort of have this infodemic…the big examples...are…ACE inhibitors and Ibuprofen… and the hemoglobin hijacking theory... All these things start and then they get amplified by social media.” (*ED physician/researcher, SG6)

HWs described some of the reasons they did not trust certain sources of information.

“*When you have some big personalities… telling people that this is the way to do things and if you're not… you're wrong and you are killing people… when you start… speaking in absolutes… I start to… lose… respect for you as an authority… I worry that their concern is about public image and not so much getting the right answer*.” (ED physician, SG8)

Social media was generally seen as unreliable because of the lack of its lack of scientific rigor.

“*I use Twitter professionally... and I actually shut it off about two or three weeks into COVID because I was...going to bed [and] I'm looking at Twitter and reading about … this person died and that person died... it was good for me to absorb...what was going on. But now I just shut Twitter off…because I'm like, ‘This is not helpful to me'… it was too anecdotal.”* (Psychiatrist/researcher, SG9)

Many physicians relied on peer-reviewed journals and their medical societies for up-to-date accurate information. However, several also expressed concern that the quality of the studies published and the lack of understanding about “preprints” (papers reporting results of studies that have not been peer reviewed) were problematic.

“*It's been an interesting time in research because part of the [misinformation] problem are these preprint servers… they're not peer reviewed… I see many go straight through to other journals without significant changes even though there should be… it's a reminder that research is very much flawed…”*(ED physician/researcher, SG6)

#### Rules of the (Capitalist) Game

One HW contrasted health care and public health systems' financial models and cultures, in the context of trying to understand the federal government's response.

“*Public health just doesn't really have a business model. It has a budget. It comes to them from tax dollars,...or grant…and health care does not think like that… within public offices, especially at the federal level, the … the understanding of how corporate health care is, is shockingly absent. Public sector folks come from public health departments. They're like do-gooders... and I say ‘Have you ever been inside a hospital... ever… had to cut budgets because you wanted to maximize margins to recruit a high-powered neurosurgeon?' and they're like, ‘What are you talking about?”'* (ED physician/administrator, SG2)

Some HWs felt that the U.S.'s capitalist political economy was not structured to mount an effective response.

“…* because there was no coordinated federal response, we were bidding against other hospitals, bidding up the price of PPE... in this super dysfunctional way… there are times when capitalism really doesn't work and this is one of them.”* (Hospitalist, SG1)

“*One thing that bothered me was that we had this big meeting… where he had all the different CEOs of different companies come on and talk about what they were going to do. It felt like such a capitalist take*.” (Pharmacy technician, EC3)

Another HW felt, similarly, that the U.S. response was what one would expect based on institutional structures.

“*Don't hate the player, hate the game – I see most of us, all of us, as just responding to the rules of engagement and the incentives that are outlined for us, right?... You can bellyache about how the rules of the game are not what you wish they were or you can try to rewrite the rules of the game.”* (ED physician/administrator, SG2)

### Theme 2: Working Under Fire

HWs discussed changes in their work since the surges began and the work-related challenges that they faced. Subthemes included: (1) The Myth of the Health Worker Hero; (2) Changes in Clinical Practice; (3) Organizational Leadership; and (4) Work Related Worries, Fears, and Loss. This major theme described HWs experiences that illustrated the toll the pandemic, government response, and citizens' behaviors was taking.

#### The Myth of the Health Worker Hero

A few physicians expressed frustration with society's framing of HWs as “heroes” and the unfair expectations they felt society had of HWs:

“*I'm gonna say that this [public applauding of HWs] has been seen as a positive… I think it's a really big negative. This whole… hero worship thing. I think it's nice that people are appreciating their nurses and their doctors and their health care providers…but I also think that this is sort of the problem… we just expect people [HWs] to fix things. We want a hero to come with their superpowers and just make it all better… we were telling people to stay home if you're sick… just wash your hands… and we got to a point, nobody wanted to listen and we have this whole, consequence because of it and now, we want our [HW] heroes to come in and clean up our mess. It just doesn't work that way. It just doesn't.”* (ED physician, SG8)

“*I'm thinking about this one tweet. Early on, a nurse said she was taking a break from her job because she had an underlying health condition… People were attacking her, being like, oh you signed up for this. Yes, she signed up to care for people but under the caveat that she'd be provided the proper PPE and she wouldn't have to risk her own life*.” (Pharmacy technician, EC3)

Some HWs also were puzzled, frustrated, or upset by factions of the public claiming the pandemic was not “real” and that people were not taking the pandemic seriously.

“*To see others not treating it [COVID] as a threat is disrespectful to not only… my family* [who are HWs]*, but other workers and essential workers*.” (Pharmacy technician EC3)

#### Changes in Clinical Practice

“*It's completely disrupted our normal way of practicing medicine.”* (ED physician, NP1)

When HWs talked about their experiences at work, much of the discussion centered on positive aspects of their experience and solidarity among coworkers. There were recommendations for continuing some of the changes in clinical practice and hope that they would result in long-lasting improvements.

“*[I] think that our teamwork in the ED is always fantastic… techs, nurses and EMS…but right now…we have a super appreciation because you're in a PPE room… and you have to do everything … to kind of spare people [co-workers] having to come in… I think that culture of collaboration, which we typically have been good at… is even enhanced and I would like to see that go forward.”* (ED physician/researcher, SG6)

Some also discussed support from the community.

“*... I know that a lot of local universities as well as hotels have been offering their spaces for health care workers that don't want to stay at their homes… to prevent transmission*.” (Pharmacy tech, EC3)

Some HWs welcomed the increased use of telemedicine and the flexibility it provided,

“*...[telemedicine] takes away...all these other barriers… people have huge transportation issues and can't get to their appointments or they don't feel like rolling out of bed and leaving their house to take… a half-hour bus ride to come here to see their therapist. They can actually have a conversation with their therapist while they're lying in bed. I mean people who are depressed, who are agoraphobic, who have anxiety leaving the home, … [telemedicine] takes away all of that.” (OUD treatment services manager, KC1)*

while others felt telemedicine presented new challenges and potential inequities in care delivery.

#### Organizational Leadership

Several HWs discussed how the response of leadership in their hospitals or organizations affected their experience with the pandemic surge. Some comments were positive,

“*The director is a very passionate but very direct person, a very practical person. Her supervision style has completely changed through this to be much more in tune with how the employees are doing mentally, much more in tune with our self-care, telling us it's okay to say no*.” (OUD treatment coordinator, KC7)

and some were negative.

“*They [workplace supervisors] are like, ‘… you're not gonna wear N95 masks. You don't need to wear it.' Really? Okay. Tell that to the nurse who didn't wear an N95 mask and ended up with COVID.”* (Hospital-based social worker, MF2)

“… *they [administrators] were… mandating things that weren't feasible and forcing employees to … not do something because it wasn't really possible … [or] be insubordinate in order to actually do their job. … people who work in administration don't want to defer to people who do the actual work*.” (OUD treatment coordinator, KC3)

#### Work-Related Worries, Fear, and Loss

HWs shared a broad range of work-related concerns in relation to the pandemic. Categories within this subtheme included health disparities and vulnerable populations, foregone health care, loss of personal connection with patients, loss of trust in the health care system, financial concerns, and provider burnout.

Worry about the pandemic's impact on people in vulnerable populations and the associated emerging racial/ethnic and socioeconomic disparities in who was contracting and dying from COVID-19 was among the most commonly discussed concerns.

“*… you got a white collar worker who can still work at home…it's still stressful… but nobody knows what other people are dealing with… I feel like it's going to be very easy to really not know how much other people are struggling…I think it has the potential to really worsen inequality in the country, but… sort of quietly and invisibly worsen it.”* (ED physician/researcher, SG7)

“*I… think it's really challenging for our homeless population… I spoke to someone [known to ED staff and homeless] at the beginning of everything, and he [said], ‘That's okay [that public buildings with restrooms were closed], I'm just not going to eat so that I don't have to use the bathroom.”'* (ED physician/researcher, SG7)

Many HWs also worried about patients forgoing health care due to fear of contracting COVID-19 if they went to a health care facility.

“*I see that a lot of people aren't coming to the hospital for preventive things. There's been a spike in people experiencing strokes and heart attacks because people aren't getting the treatment they need… there is that fear of going to the emergency room*... *people are going to go months, if not years, without getting their proper dental checkups or primary care checkups or eye checkups – that's going to create years and years of damage for people. That's going to create more strain later on*.” (Pharmacy technician, EC3)

Some HWs spoke about the diminished opportunity for personal connection when interacting with patients while gowned, masked and gloved.

“*… one more drastic difference in my day-to-day life is that my ability to connect with patients when I am wearing a ridiculous amount of gear and they cannot see my face and I cannot see their face is terrible.”* (ED physician/researcher, SG7)

Some HWs also expressed concern about loss of trust in health care systems due to pandemic-related rumors and misinformation.

“*…it did feel…sad… that there were people saying that if you had coronavirus, that we would not do CPR [at hospital x]… EMS had heard this and stopped bringing patients to our hospital for a short period of time… I wonder what's gonna happen with people and their trust in the medical community. Are they gonna feel like the hospital is super dangerous and then never ever come back?...the effects of this are gonna linger*…”(ED physician, SG8)

Concern for the financial stability of health care systems made some HWs worry about job security.

“*The hospital isn't doing well [financially]. It wasn't doing well before this…I think there's going to be a lot of people being laid off…”* (Medical records staff, MF5)

Finally, some HWs also worried about the long-term mental health effects working in the pandemic conditions would have on HWs, including the possibility of having to decide on allocation of scarce resources such as ventilators.

“*... moral distress… we talk about this in the emergency room, with situations where you know what should be done and you can't do it... if you're in a place where there's not enough ventilators and you want to put someone [on a ventilator but can't unless you take someone else off]...I think that that sort of thing is going to affect health care workers all over the country*. (ED physician, SG8)

### Theme 3: The Political Becomes Personal

HWs described both positive and negative impacts of the pandemic on their personal lives, noting often that personal effects were closely related to work effects. Subthemes for this major theme included: (1) Health Workers as Unintended Threats to the Public's Health; (2) An Emotional Toll; (3) Family: Disruptions and Silver Linings. These subthemes were largely related to Personal and Interpersonal SEM levels. Several physicians prefaced their discussion of how the pandemic was affecting them with comments about awareness of their socioeconomic privilege.

#### Health Workers as Unintended Threats to the Public's Health

Although the services HWs provided were often crucial to fighting the pandemic, some worried that their high risk of contracting COVID-19 made them a risk to patients and public health. This concern was compounded by the lack of reliable, rapid testing in the U.S. early in the pandemic. This may have contributed to the moral hazard experienced by health workers, whose work is intended to improve health and wellness and cause no harm.

“*I'm concerned with being able to get tested because I guess the fear is, jeez, what if I have the virus but I'm asymptomatic and I give it to someone else?”* (Chiropractor, EB2)

#### An Emotional Toll

Many HWs described ways in which their experiences as a HW during the pandemic were negatively affecting their physical and emotional health and the impact of their work on the mental health of their children.

“*… my [own] sobriety has probably never been shakier than it has been during this time.… I … drove by a package store for 45 minutes. Back and forth having conversations in my head, ‘Who will know? What does it matter?' Luckily, I was up to the task, and it was just … a waste of gas and time, but I can definitely understand people with less momentum [with recovery] struggling even harder, because I wasn't reaching out and asking for help. I haven't been going to in-person meetings, so I don't get to see these people and let them know how I'm doing either.”* (OUD treatment coordinator, KC7)

“* [my] 10 year old [has] told me many nights she just has a hard time turning her brain off because she's worried about people… an adjustment is having these big conversations with her, grownup conversations…people are dying.”* (Nurse practitioner, RJ3)

#### Family: Disruptions and Silver Linings

The personal impacts that HWs described were often closely related to their family situation. For example, all public schools and many private schools in New York and Massachusetts had abruptly transitioned to on-line remote learning in mid-March and most daycares closed at this time, meaning HWs who were also parents were faced with needing to care for young children and supervise older children doing remote schooling while working.

In addition to worrying about putting the public at risk because of HWs' high risk of exposure to COVID-19, HWs also worried more about the risk they put their families at than the risk to themselves.

“*I know the risk associated with being an emergency physician but I signed up for those risks… that's what I do, that's part of my job. My family didn't… it's an emotional toll for all of us with a concern that we may bring this home to our family members*.” (ED physician, NP1)

Some HWs also found that lockdown had positive aspects for their family.

“…*it's like if you have a closet and... you're thinking of throwing things out of your closet ‘cause you wanna simplify things. But...if you have something that you bought and… that stuff is really nice and you used to wear it. You're like, you don't wanna get rid of it'cause you bought it and it's hard to let it go. But when you come into an empty closet, you could just buy what you wanna buy. You fill it with what… you're gonna wear and what you wanna have. So now it's like someone just came in and… cleaned out the closet. And now I can… add stuff back as I want ‘cause I'm not… trying to empty out a really full closet*.” (Psychiatrist/researcher, SG9)

Others found it difficult to get work done while juggling home responsibilities and that increased work demands reduced the time they spent with their families.

“*I've always been kind of a workaholic but… week two of March through …the first week of April, I worked at least 18 hours a day… minimum… I'm actually spending the least amount of time with my kids… [than I have ever spent] in their whole lives...”* (ED physician/researcher, SG6)

Some HWs found themselves questioning the importance of some of the non-clinical aspects of their work and the ways in which they had previously structured their days, with some hoping to preserve some of the slow down once lockdowns and social distancing requirements ended.

“*…probably for at least a month I was very scatterbrained and I was just like… first of all, who cares about [focus of HW's research] right now, you know?... Before this [interview] I had two meetings, one with primary care and one with surgeons about [research].... I was like ‘I do not give a shit'… it's not relevant right now…maybe it will be relevant again.”* (ED physician/researcher, SG7)

### Theme 4: Hope

In addition to silver linings discussed by HWs, some also described hopes that the pandemic could result in broader positive societal changes. The concepts related to this theme centered on Societal, Organizational and Community SEM levels.

“*I feel like if this country can use the pandemic to… [institute] paid sick leave, to pass policies that should have existed and should have been in place long ago… that could be a lovely silver lining…that we say, ‘Oh, we actually need to take care of everyone with these things rather than every man for themselves.”'* (ED physician/researcher, SG7)

Some HWs hoped that the chaotic and fragmented response by the government and the health care system might advance discussions about addressing problems, big and small, in the health care system.

“*I am hopeful that the health care system will… in light of the PPE issue… think more about waste…like the amount of stuff we throw out that could be reusable*.” (ED physician/researcher, SG7)

### Interpretations in Relation to the Social Ecological Model

“*Man is an animal suspended in webs of significance he himself has spun.”*– Clifford Geertz (34)

**Figure 1:**
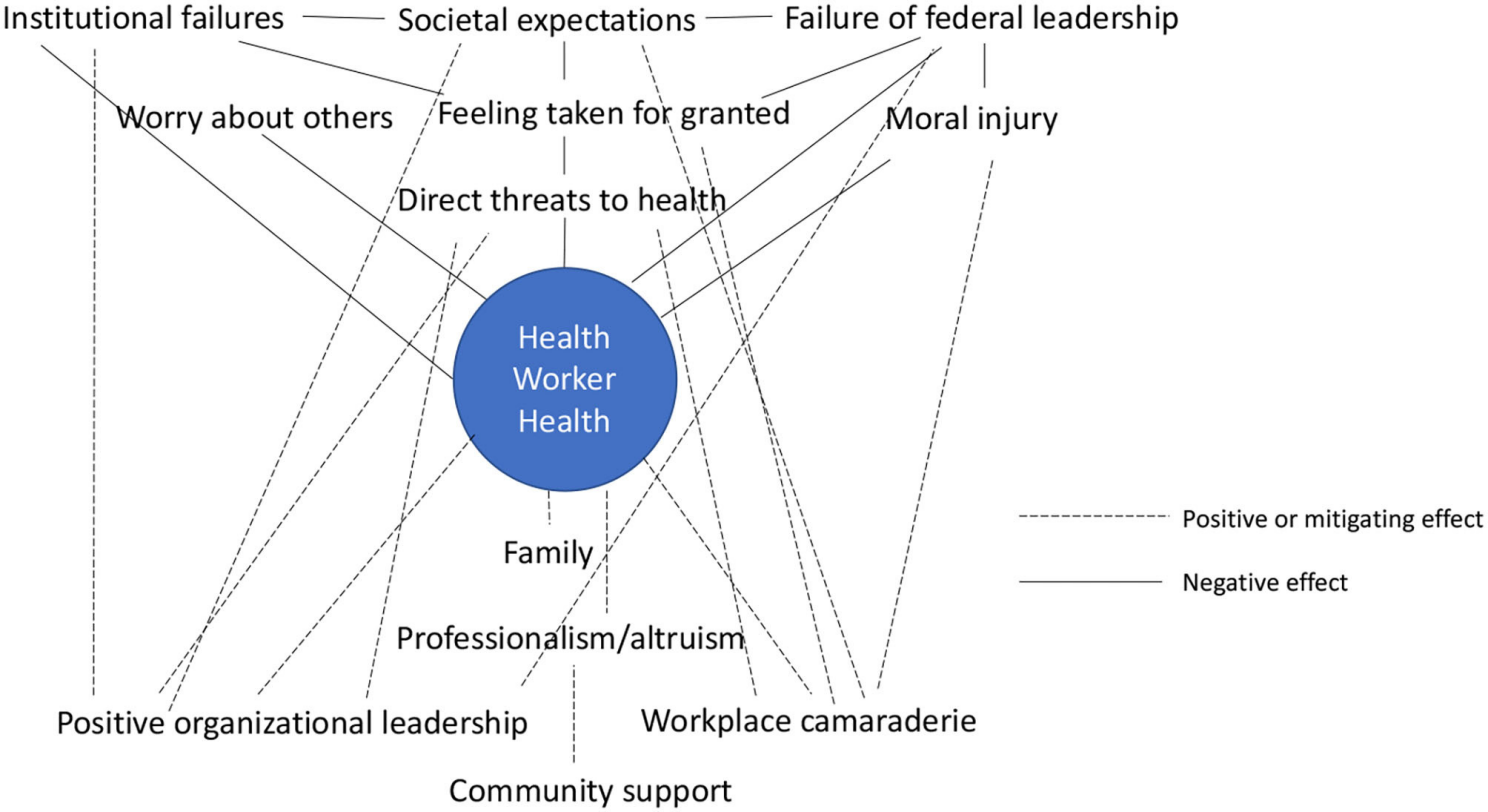
Positive and negative influences on Health worker health across social ecological levels.

The major themes and subthemes identified can be interpreted as a web of interactions involving the SEM levels (Figure 1). In the U.S., historical social, cultural, and political phenomena have generated a strongly individualistic society with a largely unregulated capitalist political economy (35). The health care and public health systems that developed within these socio-political boundaries are largely siloed and divorced from each other (36). As one HW noted, these two systems in general, and unregulated capitalism in particular, are poorly designed for addressing pandemic challenges. The absence of effective federal leadership may have made it all the more challenging to overcome the limitations of the U.S.'s sociopolitical system design, or as one HW framed it, the “Rules of the Game”. Despite the obstacles these “Rules” can present, the thoughts and experiences HWs participating in this study described suggested how actions taking place at organizational, personal, and interpersonal levels may mitigate the effects of failures at societal and structural levels.

## Discussion

The COVID-19 pandemic's rapidly changing landscape has presented political, public health, medical, community, and individual challenges. Understanding the acute and long-term occupational health effects on HWs will likely require a diverse set of research methodologies. This study was one of the first to take an in-depth qualitative exploration of HWs experiences, feelings, and perceptions during the first surges of COVID-19 infections in the U.S. While much has been learned about the clinical and epidemiological aspects of the COVID-19 virus since data for this study were collected, narrative data such as reported in this study provide the context needed to address the social complexities of the pandemic (37). The U.S. continues to lack a coordinated federal response and public health mitigation strategies such as mask-wearing and vaccination have become deeply politicized, meaning that HWs' heavy workloads, isolation, anxiety, grief, and death are likely to continue for some time. As the virus spreads unabated across the nation and overwhelms health care systems, the narratives shared by the HWs who participated in this study offer insights into the potential short- and long-term impacts the pandemic may have on HWs' physical and mental health as well as potential approaches to mitigating risk. The themes identified in this study illuminate ways in which institutional and infrastructural failures have likely played a role in the U.S.'s pandemic experience but also showed sources of community, interpersonal and personal resilience, resourcefulness, and hope.

HWs in the U.S. have long had some of the highest rates of job-related stress, burnout, and suicide (38, 39). Prior infectious disease epidemics, such as SARS and Ebola, demonstrated the disparate mortality, physical, and mental health effects such epidemics can have on HWs (40). The COVID-19 pandemic is affecting HWs across the globe on a scale not seen for more than a century. In the U.S., poor public health preparedness, lack of a coordinated national response, and failure of the federal government to act swiftly using the best scientific data available may have put HWs at even greater risk for physical and mental health sequelae than they might have otherwise experienced. A study led by the National Nurses Study and the Kaiser Foundation published in a special report by the National Academies of Science, Engineering, and Medicine described how the nation's lack of a uniform system to collect, collate and report illnesses and deaths of HWs related to COVID-19 impairs the ability to accurately monitor and develop interventions to mitigate HWs' risks (41). The report calls for a national system to track not only deaths directly due to work-related COVID-19 infection but collateral deaths, such as suicides due to the fatigue, stress, and burnout, and mental health morbidity. The report notes that accuracy of reporting improved significantly for nursing homes after the Centers for Medicaid and Medicare Services (CMS) implemented a new reporting policy in May of 2020 that included penalties for failure to comply, demonstrating that accurate collection of these data is possible. The results of the current study suggest that development of a robust system to rapidly track the effects of the pandemic and identification of best practices to mitigate the pandemic effects on HWs should be a national priority. These suggestions and other interventions suggested by this study, are consistent with the World Health Organization's *Maintaining essential health services: operational guidance for the COVID-19 context*, which outlines 10 operational strategies for maintaining essential health services, which involves protection of health workers' physical and mental health (42).

The themes identified in this study raise questions about the role of HWs in society and HWs' ethical responsibilities. While some HWs felt that the work-related risks they were experiencing were part of “what they signed up for,” others questioned whether their responsibility extended to putting their lives and their families' at risk. This questioning may have been magnified by the perceived lack of support for HWs evidenced by the federal government undermining public health messages about mitigation, failure to help procure adequate PPE, and large portions of the population electing not to wear masks, practice social distancing, or, once vaccines became available, to be vaccinated. Although nurses and other staff have unionized in some regions of the country, a large portion of health workers in the U.S are not part of a labor union. Working conditions during COVID have renewed interest in health worker organization (43, 44). Physicians historically have not been thought of as workers requiring labor protection, but as employment arrangements have shifted so that more physicians are employed by hospital systems than working in private practice, the pandemic experience may raise questions as to whether there may be a role for more extensive labor organization in the future across all HW roles.

Even in the face of major stressors, themes of HW professionalism, caring, and hope emerged. A narrative review of resilience strategies to manage psychological distress among health care workers during the COVID-19 pandemic published in June 2020 suggested several approaches that tie to the current study's themes (45). For example, organizational justice and organizational strategies, including staff feedback sessions and demonstrating support for workers, link to the Organizational Leadership subtheme in the current study. It remains unclear what the widespread disregard for HWs' and their families' health and mental health may mean for sustaining an adequate workforce in some health professions or how to make clear the toll the behaviors that suggest lack of regard has had.

### Limitations

The results of this study should be considered in the context of its limitations. First, one of the goals of the study was to interview HWs in the midst of the first surges in the country. Because the duration of surges in Massachusetts and New York could not be predicted at the time and it was not certain other surges would follow, we relied on a convenience plus snowball sampling approach to recruit participants. This allowed us to recruit HWs quickly and include HWs with a diversity of roles in health care. This also meant that the HWs who were interviewed by someone they knew may have been more or less willing to share controversial or critical thoughts but the criticisms and difficult topics discussed by HWs suggest familiarity may have facilitated openness. The majority of HWs interviewed were white; interviews with Black, Latinx and otherwise socially marginalized HWs as well as HWs in the lowest paying HW jobs may have generated additional themes or alternative views of the themes identified. Interviewers were affiliated with a university in a state with a tradition of liberal politics. Although political affiliation data were not collected, the political divisions in the country may have meant that different perspectives may be obtained in states that experienced surges later in the course of the pandemic with differing majority political views. Some questions in the interview guide were tailored to the earliest days of COVID-19 spread and may have less relevance at the current stage of the pandemic. Although the interviews for this study were conducted in the beginning of the pandemic, the mental health and physical consequences affecting HWs are likely to continue and potentially worsen as infection and death rates continue to climb.

## Conclusions

This study of U.S. HWs experiences in the early days of the COVID-19 pandemic generated important narrative insights into the unique physical and psychological risks to HWs and similarities to risks identified in prior serious respiratory viral infection epidemics such as SARS-CoV. Although little of the response to the pandemic to date has involved a coordinated effort at the federal or other level, it is of urgent importance that the health and well-being of HWs be protected. The potential need for change at multiple levels of the SEM that were suggested by this study that could be tested in a large representative sample of health workers. For example, at the Societal level, combining the national databases tracking health worker infection rates, morbidity and mortality, coordinating efforts to implement evidence-based protective interventions in health care settings while also trying to understand address the forces that have reduced concern for the collective good and addressing the problematic capacity for emergency response are high level needs. At the Organizational level, management training and guidelines for rapid assembly and performance of an incident command center may help support the coordination needed to protect health workers. Finally, making mental health care support and options for family support more readily accessible and affordable could potentially offer better support at the individual level.

## Data Availability Statement

Raw data will be made available on reasonable request with any data that may risk loss of confidentiality redacted.

## Ethics Statement

The studies involving human participants were reviewed and approved by University of Massachusetts, Amherst. The Ethics Committee waived the requirement of written informed consent for participation.

## Author Contributions

SG conceived of the study, trained team members, interviewed participants, led the analysis, and drafted the manuscript. KC, MF, EC, and NP made intellectual contributions to study development, interviewed participants, participated in the analysis, contributed to manuscript review and editing, and approved the final version. MD and KW made intellectual contributions to study development, participated in the analysis, contributed to manuscript review and editing, and approved the final version. All authors contributed to the article and approved the submitted version.

## Conflict of Interest

The authors declare that the research was conducted in the absence of any commercial or financial relationships that could be construed as a potential conflict of interest.

## Publisher's Note

All claims expressed in this article are solely those of the authors and do not necessarily represent those of their affiliated organizations, or those of the publisher, the editors and the reviewers. Any product that may be evaluated in this article, or claim that may be made by its manufacturer, is not guaranteed or endorsed by the publisher.
